# Neuronal protein with tau-like repeats (PTL-1) regulates intestinal SKN-1 nuclear accumulation in response to oxidative stress

**DOI:** 10.1111/acel.12285

**Published:** 2014-11-14

**Authors:** Yee Lian Chew, Jürgen Götz, Hannah R Nicholas

**Affiliations:** 1 School of Molecular Bioscience, University of SydneySydney, NSW, Australia; 2 Clem Jones Centre for Ageing Dementia Research (CJCADR) at the Queensland Brain Institute (QBI), University of QueenslandBrisbane (St Lucia campus), QLD, Australia

**Keywords:** *C. elegans*, lifespan, neurons, oxidative stress, PTL-1, SKN-1

## Abstract

Oxidative stress is a central pathomechanism in Alzheimer's disease (AD) and other diseases with tau pathology. The Nrf2 transcription factor induces detoxification enzymes and improves tau pathology and cognition. Its homologue in *C. elegans* is SKN-1. We previously showed that the worm tau homologue, PTL-1, regulates neuronal aging and lifespan. Here, we tested PTL-1's involvement in the stress response. *ptl-1* mutant animals are hypersensitive to oxidative stress and are defective in stress-mediated nuclear accumulation of SKN-1. This defect can be rescued by PTL-1 re-expression under the control of the *ptl-1* promoter. Given the close relationship between aging and stress tolerance, we tested lifespan and found that PTL-1 and SKN-1 regulate longevity via similar processes. Our data also suggest that PTL-1 functions via neurons to modulate SKN-1, clarifying the role of this protein in the stress response and longevity.

## Introduction, results and discussion

The most common form of dementia, AD, is characterised by Aβ-containing plaques and neurofibrillary tangles composed of hyperphosphorylated tau (Ittner *et al*., 2011). Protein with tau-like repeats-1 (PTL-1) is the sole *Caenorhabditis elegans* homologue of the mammalian tau/MAP2/MAP4 family (Goedert *et al*., 1996). PTL-1 has a predominantly neuronal expression pattern and functions in the nervous system to mediate kinesin-based transport (Tien *et al*., 2011).

Activation of Nrf2, a mediator of the oxidative stress response, reduces tau hyperphosphorylation and aggregation (Jo *et al*., 2014; Stack *et al*., 2014). Its *C. elegans* homologue, SKN-1, similarly regulates an oxidative stress response (An *et al*., 2003). SKN-1 exists in 3 isoforms (Bishop *et al*., 2007). Most studies have focused on isoforms b and c, and a SKN-1b/c::GFP transgenic line is available, facilitating expression studies (An *et al*., 2003). SKN-1b mediates dietary-restriction-mediated longevity (Bishop *et al*., 2007) and SKN-1b/c re-expression compensates for the loss of isoforms a/c in the oxidative stress response (An *et al*., 2005).

**Figure 1:**
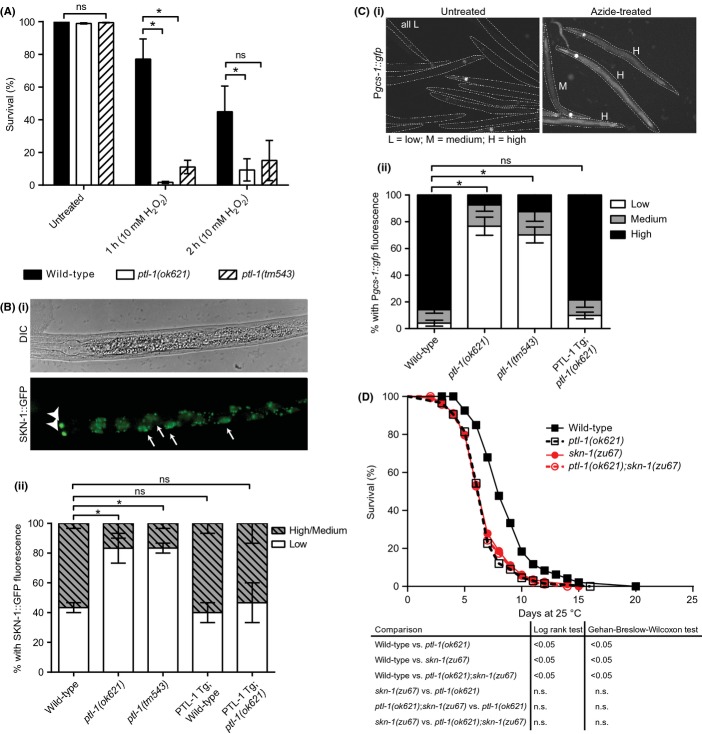
PTL-1 regulates the stress response and longevity in the same pathway as SKN-1. A) *ptl-1* mutants are hypersensitive to H_2_O_2_ stress. Bi) Intestinal *SKN-1::GFPb/c* nuclear accumulation in response to sodium azide stress is indicated by arrows pointing to intestinal nuclei. Arrowheads indicate ASI neurons. Bii) *ptl-1* mutants are defective in SKN-1 nuclear accumulation in response to azide, which can be rescued by PTL-1 re-expression. SKN-1 nuclear accumulation was scored as positive if GFP was localised to ≥ 1 intestinal nucleus. n = 15 per assay for 2 replicates. Ci) Azide induces P*gcs-1::gfp* expression. Scoring was conducted as in (Wang *et al*., 2010). Cii) *ptl-1* mutants show defective P*gcs-1::gfp* induction in response to azide, which can be rescued by PTL-1 re-expression. n = 40 per replicate for 3 replicates. D) Survival curves at 25 °C. n = 120 per assay for 2 replicates (one shown). For graphs in Bii) and Cii), error bars indicate mean±SEM. p-value: *<0.05, ns=not significant. For details of statistical analysis see Experimental Procedures.

Loss of *ptl-1* causes neuronal and organismal aging defects (Chew *et al*., 2013, 2014). As aging and stress pathways are intimately linked, we tested whether *ptl-1* mutant animals are stress-sensitive. *ptl-1(ok621)* and *ptl-1(tm543)* mutant worms showed decreased survival after exposure to H_2_O_2_ (Fig 1A). We next tested whether SKN-1 was affected by defective PTL-1. Wild-type animals carrying the *ldIs7[SKN-1::GFP]* transgene show reporter expression in the cytoplasm of intestinal cells that rapidly accumulates in the nucleus in response to stress. In contrast, in ASI neurons, SKN-1 is constitutively localised to nuclei (An *et al*., 2003)(Fig. 1Bi). In the following assays, we used azide stress as this was shown to effectively induce SKN-1 nuclear accumulation (An *et al*., 2003). In nonstress conditions, no SKN-1 nuclear accumulation was observed in any of the tested strains (data not shown). Both *ptl-1* mutant strains displayed a defect in SKN-1 accumulation in intestinal nuclei in response to azide (Fig. 1Bii), which could be rescued by re-expression of PTL-1 under control of the *ptl-1* promoter (Fig 1Bii). We next tested whether GCS-1, a detoxification enzyme that is induced by SKN-1, is affected by mutations in *ptl-1*. *ldIs3[*P*gcs-1*::*gfp]* expression is low under normal conditions (Fig S1) but is induced in the intestine under stress conditions (Fig 1Ci) (An *et al*., 2003). P*gcs-1*::*gfp* induction in response to stress was defective in *ptl-1* mutants, and this defect was rescued by PTL-1 re-expression (Fig. 1Cii). We also found that the induction of two other SKN-1 targets, *gst-4 (*Park *et al*., 2009*)* and *hsp-4* (Glover-Cutter *et al*., 2013), following azide treatment was compromised in *ptl-1* mutants and could be rescued by PTL-1 re-expression (Fig S2). We previously showed that *ptl-1* mutants are short-lived (Chew *et al*., 2013, 2014). Others reported that *skn-1(zu67),* which affects SKN-1a and c, also confers a short-lived phenotype (An *et al*., 2003). Interestingly, *ptl-1;skn-1* double-mutant animals did not have a significantly different lifespan compared to *skn-1* or *ptl-1* single-mutant animals (Fig 1D, Table S1), suggesting that SKN-1 and PTL-1 regulate lifespan via the same pathway.

**Figure 2:**
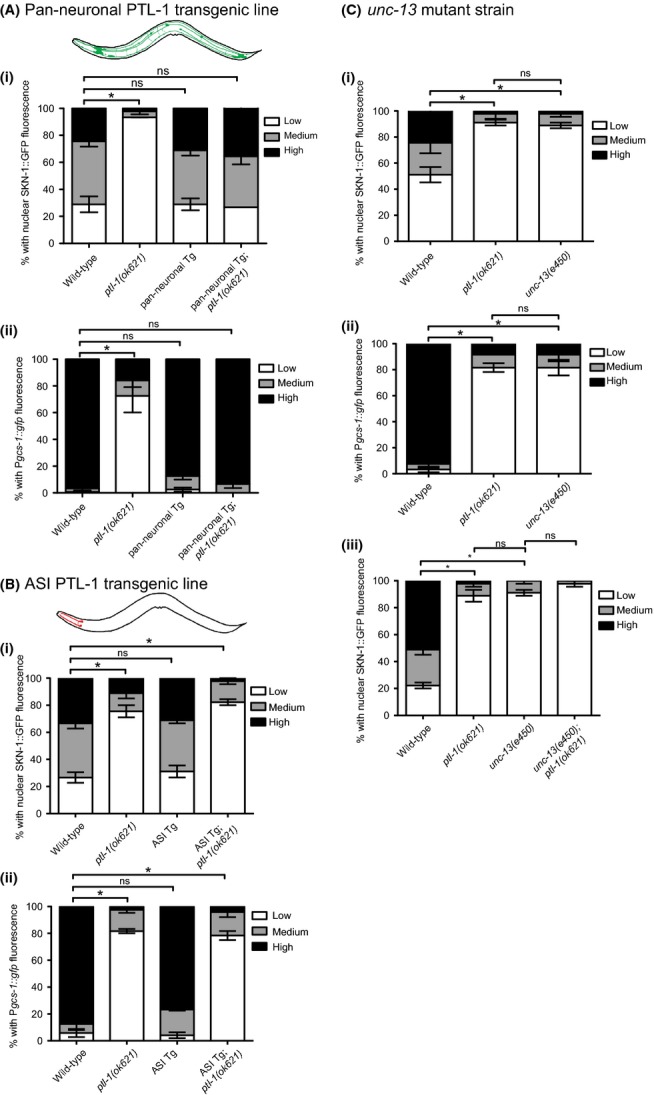
Neuronal PTL-1 regulation of intestinal SKN-1 may involve UNC-13. A) Pan-neuronal re-expression rescues the defect in i) intestinal SKN-1 nuclear accumulation and ii) P*gcs-1::gfp* induction in response to azide. B) ASI neuron-specific re-expression fails to rescue the defect in i) intestinal SKN-1 nuclear accumulation and ii) P*gcs-1::gfp* induction in response to azide. C) *unc-13* mutant animals are defective in i) intestinal SKN-1 nuclear accumulation and ii) P*gcs-1::gfp* induction in response to azide. iii) intestinal SKN-1 nuclear accumulation for *unc-13;ptl-1* double-mutant animals treated with azide. Scoring was conducted as in (Tullet *et al*., 2008; Wang *et al*., 2010). For SKN-1::GFP, n = 15 per assay for 3 replicates; for P*gcs-1::gfp*, n = 40 per assay for 3 replicates. Error bars indicate mean±SEM. p-value: *<0.05, ns=not significant. For details of statistical analysis, see Experimental Procedures.

Using the pan-neuronal *aex-3* promoter, we re-expressed PTL-1 to test whether neuronal PTL-1 regulates SKN-1. This was sufficient to rescue the defect in sensitivity to H_2_O_2_ (Fig S3), SKN-1 nuclear accumulation (Fig 2Ai), P*gcs-1*::*gfp* induction (Fig. 2Aii) and induction of *gst-4* and *hsp-4* (Fig S2) in *ptl-1* null mutants in response to stress. These data suggest a role for neuronal PTL-1 in regulating intestinal SKN-1. However, as *aex-3* is also reported to function in the intestine (Mahoney *et al*., 2008), a contribution from non-neuronal tissues to the observed rescue of *ptl-1* mutant phenotypes cannot be excluded. We therefore also performed RNAi knockdown of *ptl-1* and found that SKN-1 nuclear accumulation in response to stress is only compromised when the nervous system is sensitised to RNAi, supporting a role for neuronal PTL-1 in intestinal SKN-1 regulation (Fig S4). Given that SKN-1b is expressed in ASI neurons (An *et al*., 2003; Bishop *et al*., 2007), we tested whether re-expressing PTL-1 in ASI neurons alone, using a *gpa-4* promoter, affected intestinal SKN-1. However, ASI-specific PTL-1 re-expression neither rescued the defect in SKN-1 nuclear accumulation nor enabled P*gcs-1::gfp* induction (Fig 2B). These findings suggest that PTL-1 in the nervous system, but not ASI neurons alone, modulates SKN-1 accumulation in the intestinal nuclei in response to stress. PTL-1 may be required for communication between neurons and the intestine, via synaptic vesicle (SV) transport. We found that *unc-13(e450)* mutants that are defective in SV exocytosis (Richmond *et al*., 1999) are also defective in SKN-1 nuclear accumulation and P*gcs-1::gfp* induction in response to azide stress (Fig 2C). When we generated a *unc-13;ptl-1(ok621)* double-mutant strain, we did not observe differences in SKN-1 localisation between azide-treated *unc-13* and *unc-13;ptl-1* strains (Fig. 2Ciii), suggesting that PTL-1 and UNC-13 act in the same pathway to regulate SKN-1.

We have shown that the tau-like protein PTL-1 is involved in regulating the response to oxidative stress and in regulating aging, likely in the same pathway as SKN-1. In addition to DAF-2 (Tullet *et al*., 2008) and p38 MAPK (Inoue *et al*., 2005), we propose PTL-1 as an additional factor required for nuclear localisation of intestinal SKN-1. We did not find a role for insulin signalling in PTL-1-mediated SKN-1 regulation (Fig S5). As PTL-1 regulates kinesin-based transport (Tien *et al*., 2011), neuronal PTL-1 may regulate intestinal SKN-1 via signalling molecules carried by SVs. In support, we showed that SKN-1 nuclear accumulation requires UNC-13. Interestingly, *unc-13* expression may be regulated by SKN-1(Staab *et al*., 2014).

Our data contribute to an emerging picture of a complex communication network between the nervous system and the intestine in *C. elegans*. Related to this is work on the SKN-1 negative regulator WDR-23, which is widely expressed and targets SKN-1 for proteasomal degradation (Choe *et al*., 2009). Intestinal WDR-23 expression is sufficient to rescue the neuromuscular defect in *wdr-23* mutant animals (Staab *et al*., 2013), implying that intestinal SKN-1 regulates neuronal function.

We previously showed that PTL-1 regulates neuronal and organismal aging (Chew *et al*., 2013, 2014), and now show that it modulates the stress response via SKN-1. Given that tau pathology has also been linked to oxidative stress, our findings provide an interesting avenue for a further investigation into the role of a tau-like protein in stress tolerance and longevity.

## Supporting Information

Supplementary text Including Supplementary Results and Discussion, Experimental Procedures and Supplementary Figure legends.

**Fig. S1** Non-stressed animals display a low basal induction of P*gcs-1::gfp*.

**Fig. S2** Induction of SKN-1 targets *gst-4* and *hsp-4* is impaired in *ptl-1* mutant animals and can be rescued by PTL-1 re-expression.

**Fig. S3** Re-expression of PTL-1 under the control of the *ptl-1* promoter or the *aex-3* promoter restores resistance to hydrogen peroxide treatment.

**Fig. S4** Knockdown of *ptl-1* results in defective SKN-1 nuclear accumulation when neurons are sensitised to RNAi.

**Fig. S5** PTL-1 does not regulate DAF-16::GFP nuclear localisation, and SKN-1 nuclear accumulation is not affected by mutations in *daf-2*.

**Table S1** Summary of data obtained in two independent lifespan assays for *ptl-1(ok621), skn-1(zu67)* and *ptl-1(ok621);skn-1(zu67)* strains together with a wild-type control.
