# Supplementary material for: Neuronal protein with tau-like repeats (PTL-1) regulates intestinal SKN-1 nuclear accumulation in response to oxidative stress
Source: Aging Cell. 2014 Nov 14;14(1):148–51. doi: 10.1111/acel.12285 (PMC4326904; doi:10.1111/acel.12285)
Supplement: Supplementary file 1 — Supplementary text Including Supplementary Results and Discussion, Experimental Procedures and Supplementary Figure legends. [file acel0014-0148-sd1.docx]

**Supplementary information**

**Results and discussion**

The insulin-like signalling pathway is one of the most well-understood regulators of longevity and stress tolerance([Kenyon *et al.* 1993](#_ENREF_9)). The insulin receptor DAF-2 was shown to regulate SKN-1 accumulation in intestinal nuclei([Tullet *et al.* 2008](#_ENREF_15)), and SKN-1-mediated regulation of lifespan may involve DAF-2 and its downstream effector DAF-16([Robida-Stubbs *et al.* 2012](#_ENREF_11)). We investigated if DAF-2 signalling via DAF-16 was also affected in *ptl-1* mutant animals. Like SKN-1, DAF-16::GFP accumulates in intestinal nuclei in response to oxidative stress (**Fig S5Ai**). This nuclear accumulation of DAF-16 is unaffected by loss of PTL-1 (**Fig S5Aii**). We next tested if DAF-2 is involved in PTL-1-mediated regulation of SKN-1. Contrary to previous data([Tullet *et al.* 2008](#_ENREF_15)), we were unable to resolve an effect of *daf-2(e1370)* mutation on SKN-1 nuclear accumulation in untreated animals or in response to oxidative stress at 15 °C or 20 °C (**Fig S5B**). In untreated animals, we did not observe any nuclear SKN-1::GFP in any of the strains, including *daf-2(e1370)* (data not shown). This discrepancy may be due to the difference in transgenic lines used here compared with the previous investigation. We used a SKN-1::GFP integrated transgenic line that expresses at a much lower level compared with the extrachromosomal line used previously. Importantly, *daf-2* mutation did not affect the defective SKN-1 response in *ptl-1* mutant animals (**Fig S5B**).

**Experimental procedures**

*1. Strain information*

*C. elegans* strains were cultured on NGM plates seeded with the *Escherichia coli* strain OP50. Hermaphrodite animals were used for all experiments. The wild-type strain used for all experiments is N2 (Bristol). Strains **N2**, **RB809** *ptl-1(ok621)*, **CB1370** *daf-2(e1370)*, **EU1** *skn-1(zu67) IV/nT1[unc-?(n754) let-?](IV;V)*, **LD1** *ldIs7*[skn-1B/C::GFP + pRF4(*rol-6(su1006)*)], **LD1171** *ldIs3*[*gcs-1*p::GFP + pRF4(*rol-6(su1006)*)], **TJ356** *zIs356*[*daf-16*p::DAF-16::GFP + pRF4(*rol-6(su1006)*)], **VP303** *rde-1(ne219); kbIs7[nhx-2p::rde-1 + rol-6(su1006)]*, **TU3401** *sid-1(pk3321); uIs69[pCFJ90(myo-2::mCherry) + unc-119p::sid-1]*, **SJ4005** *zcIs4*[*hsp-4*::GFP], and **CL2166** *dvIs19*[pAF15(*gst-4*::GFP::NLS)] were obtained from the *Caenorhabditis* Genetics Centre (CGC), and **FX00543** *ptl-1(tm543)* was obtained from the National Bioresource Project, Japan (Dr S. Mitani). The following strains were generated by us previously and were used in this work: *ptl-1* mutant lines **RB809** and **FX00543** were both outcrossed six times to wild-type and renamed **APD004** and **APD015**, respectively. **APD026** *apdIs5*[P*ptl-1*:PTL-1::V5:*ptl-1_*3ʹUTR; P*myo-2*:*gfp*; P*rpl-28*::PuroR::rpl-16_outron::NeoR::let-858_3ʹUTR], outcrossed six times to wild-type. **APD039** *ptl-1(ok621);apdIs5*. **APD096** *apdIs10*[*aex-3*p:PTL1-V5:PTL-1 3' UTR; *myo-2*p:*mCherry*; *rpl-28*p::PuroR::*rpl-16*_outron::NeoR::*let-858*_3ʹUTR], outcrossed six times to wild-type. **APD105:** *apdIs10*; *ptl-1(ok621)*.

The *ptl-1* deletion mutant allele *ok621* was previously described in ([Gordon *et al.* 2008](#_ENREF_7); [Chew *et al.* 2013](#_ENREF_4)) and *tm543* in ([Chew *et al.* 2013](#_ENREF_4)). Briefly, *ok621* is a deletion of 1933 bp that results in a null mutation ([Gordon *et al.* 2008](#_ENREF_7)). *tm543* is a deletion of 788 bp in that putatively results in a truncated mutant protein lacking the C-terminal microtubule-binding region of PTL-1.

List of strains generated:

**APD055** *ptl-1(ok621);ldIs7*. **APD059** *ptl-1(ok621);zIs356*. **APD060** *daf-2(e1370)* *ptl-1(ok621)*. **APD069** *ptl-1(tm543);ldIs7*. **APD077** *apdIs5*;*ldIs7*. **APD078** *apdIs5*;*ptl-1(ok621);ldIs7*. **APD083** *daf-2(e1370);ldIs7*. **APD084** *daf-2(e1370)ptl-1(ok621)*;*ldIs7*. **APD091** *ptl-1(ok621);ldIs3*. **APD092** *ptl-1(tm543);ldIs3*. **APD093** *apdIs5*;*ldIs3*. **APD094** *apdIs5*;*ptl-1(ok621);ldIs3*. **APD101** *apdIs10*;*ldIs7*. **APD102** *apdIs10*;*ptl-1(ok621);ldIs7*. **APD103** *apdIs10*;*ldIs3*. **APD104** *apdIs10*;*ptl-1(ok621);ldIs3*. **APD118** *unc-13(e450);ldIs7*. **APD122** *unc-13(e450);ldIs3*. **APD118** *unc-13(e450);ptl-1(ok621);ldIs7*. **APD127** *ptl-1(ok621);skn-1(zu67)* *IV/nT1[unc-?(n754) let-?](IV;V)*. **APD136** *apdEx13* [P*gpa-4*:PTL1-V5:PTL-1 3′ UTR; P*myo-2*:*mCherry*; P*rpl-28*::PuroR::*rpl-16*_outron::NeoR::*let-858*_3ʹUTR]. **APD138** *apdEx13*; *ldIs7*. **APD139** *apdEx13;ldIs7;ptl-1(ok621)*. **APD140** *apdEx13*; *ldIs3*. **APD141** *apdEx13;ldIs3;ptl-(ok621)*. **APD142** *ldIs7* *V/ uIs69 sid-1(pk3321) V*. **APD143** *ldIs7; kbIs7.* **APD144** *zcIs4; ptl-1(ok621).* **APD145** *zcIs4; ptl-1(ok621);apdIs10.* **APD146** *zcIs4; ptl-1(ok621);apdIs5.* **APD147** *dvIs19; ptl-1(ok621).* **APD148** *dvIs19; ptl-1(ok621);apdIs10.* **APD149** *dvIs19; ptl-1(ok621);apdIs5.*

*2. Generation of transgenic lines*:

The *gpa-4* promoter was amplified from genomic DNA using primers 5′ aattGCATGCgctgatttgccgtttgtcg and 5′ cttattcattttgtgaacacttttcaacaACCGGT (containing SphI and AgeI sites, respectively) then inserted by conventional restriction cloning into a pENTR vector containing attL1 and attL4 sites to generate pY014. The multisite Gateway method was then used to combine the pENTR clones pSB011 (*aex-3* promoter)([Chew *et al.* 2014](#_ENREF_5)) or pY014 (*gpa-4* promoter), pY002 (PTL-1::V5) and pY003 (PTL-1 3ʹUTR) ([Chew *et al.* 2013](#_ENREF_4)) into dual antibiotic selection destination vector pBCN40 (J Semple and B Lehner) containing visual marker *myo-2*p::*mCherry*  to generate pY011 or pY015, respectively. Transgenic worms were generated by biolistic transformation using the PDS-1000/He™ particle delivery system (BioRad) according to the manufacturer’s instructions. Wild-type worms were bombarded with 7 µg of linearised plasmid DNA using previously established methods ([Praitis *et al.* 2001](#_ENREF_10)). Selection post-bombardment was undertaken using the dual antibiotic selection protocol ([Semple *et al.* 2012](#_ENREF_12)), as previously described ([Chew *et al.* 2013](#_ENREF_4)). Lines expressing PTL-1::V5 under the control of the *aex-3* promoter ([Chew *et al.* 2014](#_ENREF_5)) and PTL-1 3ʹUTR or *gpa-4* promoter and PTL-1 3ʹ UTR were obtained and outcrossed six times to wild-type.

*3. Stress experiments*

Animals were cultured at room temperature (23 °C) and day one adults were used for all experiments. Animals were incubated with 10 mM hydrogen peroxide in M9 buffer for 1 or 2 hours at room temperature with gentle agitation. Untreated controls were incubated with M9 buffer alone for the same time period. After the incubation time, animals were plated out onto NGM agar plates and survival scored 3-4 hours post treatment.

*4. GFP localisation experiments*

Animals were cultured at 20 °C and L2 stage worms used for analysis in all experiments unless otherwise stated. To monitor changes in GFP localisation under stress conditions, animals were incubated with 50 mM sodium azide (Sigma) in M9 buffer (azide treated), or M9 alone (untreated) for 10 minutes with gentle agitation. SKN-1::GFP localisation experiments were conducted using the *ldIs7* strain (integrated SKN-1 B/C::GFP), where expression was reported to be the highest in the L2 stage ([An & Blackwell 2003](#_ENREF_1)). For **Fig 1Bii**, SKN-1::GFP intestinal nuclear localisation was scored as “high/medium” if GFP was observed in any of the intestinal nuclei, and “low” if GFP was not observed in any nuclei. For all other SKN-1::GFP assays (**Fig 2** and **Fig S2**), SKN-1::GFP nuclear localisation scored as “high” if GFP was seen in all intestinal nuclei, “medium” if GFP was observed only in anterior or posterior nuclei, or “low” if GFP was not observed in any intestinal nuclei, essentially as in ([Tullet *et al.* 2008](#_ENREF_15)). Untreated animals for all experiments showed nuclear SKN-1::GFP in the intestine (data not shown).

DAF-16::GFP localisation assays were conducted using the *zIs356* transgene (integrated DAF-16::GFP). For DAF-16::GFP localisation experiments, young adult animals incubated with 50 mM sodium azide (azide-treated) or M9 buffer (untreated) for 10 minutes at room temperature with gentle agitation were scored as positive if GFP was observed in any of the intestinal nuclei. For *daf-2(e1370)* mutant strains, animals were incubated at either 15 or 20 °C and SKN-1::GFP assays conducted as above.

For P*gcs-1::gfp* experiments, young adult animals were treated in 50 mM sodium azide (azide-treated) or M9 buffer (untreated) for 10 minutes and scored as “high” if GFP was clearly seen in the intestine, “medium” if GFP was seen in both anterior and posterior ends of the animal, or “low” if GFP was observed only around the pharynx. Untreated animals for all experiments showed no response or a very low basal response in all genotypes tested (**Fig S1**).

For P*hsp-4::gfp* and P*gst-4::gfp* experiments, young adult animals were treated in 50 mM sodium azide (azide-treated) or M9 buffer (untreated) for 10 minutes. Fluorescence intensity was quantified using Fiji software (ImageJ) by measuring the “mean grey value” of fluorescence signals using a selection from the tail up to but not including the posterior bulb of the pharynx. The background fluorescence was subtracted from these measurements. Fluorescence intensities from 15-20 animals per treatment per genotype were measured and averaged data over 2 experiments are shown.

In all experiments, samples were imaged using a BX51 Microscope (Olympus) and micrographs captured using AnalySIS software (Olympus). Experiments were conducted blind to the genotype of the strains. For all experiments, averaged data from 2-3 independent assays is shown.

*5. RNA interference experiments*

Experiments were conducted at 20 °C. LD1, APD142 or APD143 animals were synchronised by hypochlorite bleaching and larval stage 1 worms plated onto carbenicillin plates seeded with RNAi bacteria (HT115 *E. coli*) that either contained the pL4440 empty vector (EV), or pL4440 containing cloned *ptl-1* DNA generated by the Ahringer laboratory ([Fraser *et al.* 2000](#_ENREF_6)). We have used the same *ptl-1* RNAi feeding clone in previous experiments ([Chew *et al.* 2014](#_ENREF_5)) and have shown knockdown of a PTL-1::GFP fusion protein with this RNAi clone under similar experimental conditions. Bacterial cultures were induced to express dsRNA using 3 mM isopropyl β-D-1-thiogalactopyranoside (IPTG). At the second generation fed on RNAi bacteria, animals were imaged at L2 stage to score SKN-1::GFP localisation in response to azide stress as described above. Scoring was conducted essentially as in ([Tullet *et al.* 2008](#_ENREF_15)). Analysis was conducted blind to the identity of the RNAi clones.

*6. Statistical analysis:*

All statistical analysis was performed using the GraphPad Prism 6 software (GraphPad Software Inc.) or Microsoft Excel (Microsoft). α-level is 0.05 for all analyses. For GFP localisation experiments, a one-way ANOVA comparing the incidence of ‘low’ fluorescence was conducted. P-values are indicated by ns = not significant, *<0.05.

An JH , Blackwell TK (2003). SKN-1 links C. elegans mesendodermal specification to a conserved oxidative stress response. *Genes & development*. **17**, 1882-1893.

Borner K, Niopek D, Cotugno G, Kaldenbach M, Pankert T, Willemsen J, Zhang X, Schurmann N, Mockenhaupt S, Serva A, Hiet MS, Wiedtke E, Castoldi M, Starkuviene V, Erfle H, Gilbert DF, Bartenschlager R, Boutros M, Binder M, Streetz K, Krausslich HG , Grimm D (2013). Robust RNAi enhancement via human Argonaute-2 overexpression from plasmids, viral vectors and cell lines. *Nucleic acids research*. **41**, e199.

Calixto A, Chelur D, Topalidou I, Chen X , Chalfie M (2010). Enhanced neuronal RNAi in C. elegans using SID-1. *Nature methods*. **7**, 554-559.

Chew YL, Fan X, Gotz J , Nicholas HR (2013). PTL-1 regulates neuronal integrity and lifespan in C. elegans. *Journal of cell science*. **126**, 2079-2091.

Chew YL, Fan X, Gotz J , Nicholas HR (2014). Regulation of age-related structural integrity in neurons by protein with tau-like repeats (PTL-1) is cell autonomous. *Scientific reports*. **4**, 5185.

Fraser AG, Kamath RS, Zipperlen P, Martinez-Campos M, Sohrmann M , Ahringer J (2000). Functional genomic analysis of C. elegans chromosome I by systematic RNA interference. *Nature*. **408**, 325-330.

Gordon P, Hingula L, Krasny ML, Swienckowski JL, Pokrywka NJ , Raley-Susman KM (2008). The invertebrate microtubule-associated protein PTL-1 functions in mechanosensation and development in Caenorhabditis elegans. *Development genes and evolution*. **218**, 541-551.

Kamath RS, Martinez-Campos M, Zipperlen P, Fraser AG , Ahringer J (2001). Effectiveness of specific RNA-mediated interference through ingested double-stranded RNA in Caenorhabditis elegans. *Genome biology*. **2**, RESEARCH0002.0001-0002.0010.

Kenyon C, Chang J, Gensch E, Rudner A , Tabtiang R (1993). A C. elegans mutant that lives twice as long as wild type. *Nature*. **366**, 461-464.

Praitis V, Casey E, Collar D , Austin J (2001). Creation of low-copy integrated transgenic lines in Caenorhabditis elegans. *Genetics*. **157**, 1217-1226.

Robida-Stubbs S, Glover-Cutter K, Lamming DW, Mizunuma M, Narasimhan SD, Neumann-Haefelin E, Sabatini DM , Blackwell TK (2012). TOR signaling and rapamycin influence longevity by regulating SKN-1/Nrf and DAF-16/FoxO. *Cell metabolism*. **15**, 713-724.

Semple JI, Biondini L , Lehner B (2012). Generating transgenic nematodes by bombardment and antibiotic selection. *Nature methods*. **9**, 118-119.

Tabara H, Sarkissian M, Kelly WG, Fleenor J, Grishok A, Timmons L, Fire A , Mello CC (1999). The rde-1 gene, RNA interference, and transposon silencing in C. elegans. *Cell*. **99**, 123-132.

Timmons L, Court DL , Fire A (2001). Ingestion of bacterially expressed dsRNAs can produce specific and potent genetic interference in Caenorhabditis elegans. *Gene*. **263**, 103-112.

Tullet JM, Hertweck M, An JH, Baker J, Hwang JY, Liu S, Oliveira RP, Baumeister R , Blackwell TK (2008). Direct inhibition of the longevity-promoting factor SKN-1 by insulin-like signaling in C. elegans. *Cell*. **132**, 1025-1038.

Wang J, Robida-Stubbs S, Tullet JM, Rual JF, Vidal M , Blackwell TK (2010). RNAi screening implicates a SKN-1-dependent transcriptional response in stress resistance and longevity deriving from translation inhibition. *PLoS genetics*. **6**.

**Supplementary Figure and Table legends**

**Figure S1: Non-stressed animals display a low basal induction of P*gcs-1::gfp.*** Day one adult animals carrying the *ldIs3[*P*gcs-1::gfp]* reporter were assayed at the same time as the corresponding strains incubated with sodium azide (shown in **Figure 1,2**). P*gcs-1::gfp* fluorescence is shown in non-stressed animals for **A)** transgenic lines expressing PTL-1 under the control of the *ptl-1* promoter (“PTL-1 Tg”), **B)** transgenic lines expressing PTL-1 in neurons using the *aex-3* promoter (“pan-neuronal Tg”), **C)** transgenic lines expressing PTL-1 in ASI neurons using the *gpa-4* promoter (“ASI Tg”), and **D)** *unc-13(e450)* mutant animals. The graphs show averaged data from 3 independent experiments. Scoring was conducted as in ([Wang *et al.* 2010](#_ENREF_16)). Error bars indicate mean±SEM. p-value: ns=not significant. Details of statistical analysis are provided in **Experimental Procedures**.

**Figure S2: Induction of SKN-1 targets *gst-4* and *hsp-4* is impaired in *ptl-1* mutant animals and can be rescued by PTL-1 re-expression.** Strains used are **A)** *dvIs19[*P*gst-4::gfp*] and **B)** *zcIs4[*P*hsp-4::gfp]*. Panels (i) of both **A** and **B** show a representative wild-type animal expressing the respective reporter transgene, both in untreated and 50 mM azide treated conditions. Scale, 50 µm. Panels (ii) of **A** and **B** show the averaged fluorescence intensity (arbitrary units) measured using ImageJ software for wild-type, *ptl-1(ok621)* and transgenic animals re-expressing PTL-1 under the control of the *aex-3* promoter (“Pan-neuronal Tg; *ptl-1(ok621)*”) or the *ptl-1* promoter (“PTL-1 Tg; *ptl-1(ok621)*”). Scoring was conducted by quantifying the fluorescence intensity in a selected portion of the worm starting from the tail to the anterior-most portion of the intestine. n= 15-20 animals per treatment/genotype per assay for two replicates. The graphs show averaged data from 2 independent experiments. Error bars indicate mean±SEM. p-value: ns=not significant, *<0.05. Statistical analysis: 1-way ANOVA, Sidak’s post-test (GraphPad Software Inc.).

**Figure S3: Re-expression of PTL-1 under the control of the *ptl-1* promoter or the *aex-3* promoter restores resistance to hydrogen peroxide treatment.** n >100 animals were scored per treatment per genotype for each biological replicate. The graphs show averaged data from 4 independent experiments. Error bars indicate mean±SEM. p-value: ns=not significant, *<0.05. Statistical analysis: 2-way ANOVA, Tukey’s post-test (GraphPad Software Inc.).

**Figure S4: Knockdown of *ptl-1* results in defective SKN-1 nuclear accumulation when neurons are sensitised to RNAi.** Animals carrying the *ldIs7[SKN-1b/c::GFP]* reporter transgene were treated with empty vector control (EV) or *ptl-1* RNAi by feeding and nuclear accumulation of SKN-1::GFP was scored after azide treatment. **A)** Animals carrying *ldIs7* in a wild-type background. Since neurons in wild-type worms are generally refractory to RNAi ([Kamath *et al.* 2001](#_ENREF_8); [Timmons *et al.* 2001](#_ENREF_14)), RNAi treatment in this strain should result in knockdown in all tissues except for neurons. **B)** Animals carrying *ldIs7* together with the *kbIs7[*P*nhx-2::rde-1]* transgene, which drives expression of RDE-1 in the intestine. RDE-1 is the *C. elegans* primary Argonaute protein ([Tabara *et al.* 1999](#_ENREF_13)). As in the wild-type background, RNAi treatment in this strain should result in knockdown in all tissues except for neurons. RNAi in the intestine may be enhanced by expression of the *kbIs7* transgene, since cell culture experiments have shown that over-expression of human Argonaute 2 results in more efficient RNAi ([Borner *et al.* 2013](#_ENREF_2)). **C)** Animals heterozygous for *ldIs7* and for the *uIs69[*P*unc-119::sid-1]* transgene, which drives expression of the RNA channel SID-1 in neurons. Neuronal expression of SID-1 sensitises neurons to RNAi ([Calixto *et al.* 2010](#_ENREF_3)). RNAi treatment in this strain should result in knockdown in all tissues including neurons. Detailed strain information is provided in **Experimental Procedures**. n=30 per treatment (empty vector, EV or *ptl-1* RNAi) per genotype for two replicates. Experiments were conducted twice independently; averaged data from both experiments are shown. Scoring was conducted as in ([Tullet *et al.* 2008](#_ENREF_15)). p-value: ns=not significant, *<0.05 (unpaired t-test) comparing EV and *ptl-1* RNAi treatments.

**Figure S5: PTL-1 does not regulate DAF-16::GFP nuclear localisation, and SKN-1 nuclear accumulation is not affected by mutations in *daf-2*. A)** **i)** *zls356[Pdaf-16::DAF-16::GFP]* nuclear accumulation in response to azide stress. Arrows indicate intestinal nuclei. **ii)** *ptl-1(ok621)* mutant animals do not display a defect in DAF-16::GFP nuclear accumulation in response to 50 mM sodium azide treatment. Worms were scored as positive if DAF-16::GFP was localised to at least one intestinal nucleus. n=20 per biological replicate, averaged data from 3 independent experiments shown. **B)** *ptl-1* null mutation results in a defect in intestinal SKN-1::GFP nuclear accumulation in response to azide treatment in the presence of a *daf-2(e1370)* mutation at **i)** 15 °C and **ii)** 20 °C. n=15 per biological replicate, averaged data from 3 independent experiments shown. Error bars indicate mean±SEM. Scoring was conducted as in ([Tullet *et al.* 2008](#_ENREF_15); [Wang *et al.* 2010](#_ENREF_16)). p-value: *<0.05, ns=not significant. Details of statistical analysis are provided in **Experimental Procedures**.

**Table S1**: Summary of data obtained in two independent lifespan assays for *ptl-1(ok621), skn-1(zu67)* and *ptl-1(ok621);skn-1(zu67)* strains together with a wild-type control. Animals that displayed internal hatching or bursting or were lost were censored from the assay. Lifespan assays were conducted at 25 °C, with n=120 at the start of the experiment. Analysis of survival curves was undertaken using GraphPad Prism 6 (GraphPad Software Inc.).
