# Supplementary material for: Neuronal protein with tau-like repeats (PTL-1) regulates intestinal SKN-1 nuclear accumulation in response to oxidative stress
Source: Aging Cell. 2014 Nov 14;14(1):148–51. doi: 10.1111/acel.12285 (PMC4326904; doi:10.1111/acel.12285)
Supplement: Supplementary file 7 [file acel0014-0148-sd7.pdf]

Supplementary Table 1

Parameter	Strain(s)	Experiment 1	Experiment 2 (Fig 1D)
**Median lifespan**	Wild-type	7	8
	ptl-1(ok621)	6	7
	skn-1(zu67)	6	7
	ptl-1(ok621);skn-1(zu67)	6	7
**Censored subjects**	Wild-type	48	49
	ptl-1(ok621)	42	44
	skn-1(zu67)	15	31
	ptl-1(ok621);skn-1(zu67)	13	23
**Number of deaths**	Wild-type	72	71
	ptl-1(ok621)	78	76
	skn-1(zu67)	105	89
	ptl-1(ok621);skn-1(zu67)	107	97
**p-value for statistical comparisons (log-rank test)**	Wild-type vs *ptl-1(ok621)*	<0.05	<0.05
	Wild-type vs *skn-1(zu67)*	<0.05	<0.05
	Wild-type vs *ptl-1(ok621);skn-1(zu67)*	<0.05	<0.05
	skn-1(zu67) vs *ptl-1(ok621)*	Not significant	Not significant
	ptl-1(ok621);skn-1(zu67) vs *ptl-1(ok621)*	Not significant	Not significant
	ptl-1(ok621);skn-1(zu67) vs *skn-1(zu67)*	Not significant	Not significant
**p-value for statistical comparisons (Wilcoxon test)**	Wild-type vs *ptl-1(ok621)*	<0.05	<0.05
	Wild-type vs *skn-1(zu67)*	<0.05	<0.05
	Wild-type vs *ptl-1(ok621);skn-1(zu67)*	<0.05	<0.05
	skn-1(zu67) vs *ptl-1(ok621)*	Not significant	Not significant
	ptl-1(ok621);skn-1(zu67) vs *ptl-1(ok621)*	Not significant	Not significant
	ptl-1(ok621);skn-1(zu67) vs *skn-1(zu67)*	Not significant	Not significant
